# Draft Genome Sequence of Cellulose-Digesting Bacterium *Sporocytophaga myxococcoides* PG-01

**DOI:** 10.1128/genomeA.01154-14

**Published:** 2014-11-20

**Authors:** Lin Liu, Peiji Gao, Guanjun Chen, Lushan Wang

**Affiliations:** a State Key Laboratory of Microbial Technology, Shandong University, Jinan, China; b Key Laboratory of Development and Application of Rural Renewable Energy, Ministry of Agriculture, Chengdu, China; c College of Marine Science, Shandong University, Weihai, China

## Abstract

*Sporocytophaga myxococcoides*, a Gram-negative bacterium isolated from soil, is an efficient hydrolyzer of crystalline cellulose. Here, we report its draft genome sequence, which may provide important genetic information regarding the cellulolytic and hemicellulolytic enzymes that contribute to the cellulose-degrading abilities of this bacterium.

## GENOME ANNOUNCEMENT

*Sporocytophaga myxococcoides* is a yellow-pigmented, aerobic, Gram-negative bacterium isolated from the soil. It can also form cysts when conditions are not suitable for growth (1). *S. myxococcoides* can efficiently degrade crystalline cellulose, and it can utilize crystalline cellulose as its only carbon and energy source. However, the cellulose utilization strategy of *S. myxococcoides* is unclear. Previously, the genome sequences of *Cytophagaceae* family members *Cytophaga hutchinsonii* ATCC 33406 (2), *Dyadobacter fermentans* NS114^T^ (3), *Dyadobacter tibetensis* Y620-1 (4), *Fibrella aestuarina* BUZ 2^T^ (5), *Fibrisoma limi* BUZ 3^T^ (6), *Leadbetterella byssophila* 4M15^T^ (7), *Runella slithyformis* LSU 4^T^ (8), and *Spirosoma linguale* 1^T^ (9) have been published. Here, we present the draft genome sequence of *S. myxococcoides* strain PG-01, which may provide insights into the genomic basis of cellulose utilization by *S. myxococcoides*.

The genome of *S. myxococcoides* was sequenced with the Illumina MiSeq platform at Shanghai Majorbio. One 500-bp paired-end library was prepared and sequenced, generating 661.4 Mbp of raw data (read length, 677,324,046 bp). The raw data were cleaned by removing adapters, low-quality reads, and N-containing (>10%) reads. The obtained clean data (532.6 Mbp) were checked by ErrorCorrection and then merged by overlap relationships. The resultant quality-merged data were assembled by GS *de novo* Assembler version 2.8. Open reading frames were predicted using Glimmer 3.02 and then annotated using the NCBI nr, GO, STRING, and KEGG databases. rRNAs were predicted by Barrnap 0.4.2, and tRNAs were predicted by tRNAscan-SE version 1.3.1.

The draft genome consists of 48 contigs (>1,000 bp), with a total length of 5,993,600 bp; the *N*_50_ contig length is 594,330 bp, and the *N*_90_ contig length is 184,876 bp. The G+C content is 36.02%.

The *S. myxococcoides* draft genome includes 5,048 protein-coding genes, 42 tRNA genes, and 7 rRNA genes. Seventy-one genes encode glucoside hydrolases, which contribute to lignocellulose degradation. According to the KEGG pathway analysis, most genes encode proteins involved in glycolysis/gluconeogenesis, the tricarboxylic acid (TCA) cycle, the pentose phosphate pathway, and fatty acid metabolism. *S. myxococcoides* can take advantage of nitrate in the medium to synthesize amino acids that are needed for the biosynthesis of membrane proteins and extracellular proteins that can degrade crystalline cellulose. Furthermore, it has a few genes involved in fructose, mannose, and galactose metabolism, which are related to mucopolysaccharide production.

The genome sequence of *S. myxococcoides* can also reveal the genes involved in lignocellulose degradation and mucopolysaccharide production. Systematic analyses of such gene will be reported in the future.

### Nucleotide sequence accession numbers.

This shotgun genome project has been deposited in the DNA Data Bank of Japan (DDBJ) under the accession no. BBLT00000000.
